# High expression of E2F transcription factors 7: An independent predictor of poor prognosis in patients with lung adenocarcinoma

**DOI:** 10.1097/MD.0000000000029253

**Published:** 2022-08-19

**Authors:** Yu Zhang, Lan Lyu, Wei Wang, Liwei Zhang

**Affiliations:** a Xinjiang Medical University, Department of thoracic surgery, Feicheng Hospital Affiliated to Shandong First Medical University, China; b Department of Plastic Surgery, Feicheng Hospital Affiliated to Shandong First Medical University, China; c Department of Expert's Outpatient, Feicheng Hospital Affiliated to Shandong First Medical University, China; d Xinjiang Medical University, China.

**Keywords:** diagnosis, E2F7, lung adenocarcinoma

## Abstract

Adenocarcinoma is the most common pathological type of lung cancer. The E2F7 transcription factor has been confirmed to be related to the occurrence and development of a variety of solid tumors, but the relationship with the prognosis of lung cancer is still unclear. Therefore, we conducted this study to explore the prognostic value of E2F7 for lung adenocarcinoma (LUAD) patients.

In this study, we analyzed samples from the Cancer Genome Atlas (TCGA) to study the correlation between the expression of E2F7 and clinical features, the difference in expression between tumors and normal tissues, the prognostic and diagnostic value, and Enrichment analysis of related genes. All statistical analysis uses R statistical software (version 3.6.3).

The result shows that the expression level of E2F7 in LUAD was significantly higher than that of normal lung tissue (*P* = 1e-34). High expression of E2F7 was significantly correlated with gender (*P* = .034), pathologic stage (*P* = .046) and M stage (*P* = .025). Multivariate Cox analysis confirmed that E2F7 is an independent risk factor for OS in LUAD patients (*P* = .027). Genes related to cell cycle checkpoints, DNA damage telomere stress-induced senescence, DNA methylation, chromosome maintenance and mitotic prophase showed differential enrichment in the E2F7 high expression group.

In short, high expression of E2F7 is an independent risk factor for OS in LUAD patients and has a high diagnostic value.

## 1. Introduction

Lung cancer is the second most common malignant tumor in the world. In 2020, there will be approximately 2.2 million new cases of lung cancer worldwide. Lung cancer has become the leading cause of cancer deaths, accounting for about 18% of the total cancer deaths (about 1.8 million cases).^[1]^ Among them, lung adenocarcinoma is the most common pathological type.^[2]^ Early lung cancer has no obvious symptoms, so most patients are already in the advanced stage when they are diagnosed, which leads to a generally low survival rate of lung cancer patients.

The E2F transcription factor family play a key role in the occurrence and development of tumors due to its important cell functions related to cell cycle regulation and apoptosis.^[3]^ As a newly discovered member of the E2F family in recent years, unlike other family members, E2F7 has two special DNA-binding domains (DBD) in structure, lacks the binding domain to the RB protein, and does not need to bind to dimerizing proteins to enter the nucleus.^[4,5]^

E2F7 is a priming factor involved in cell cycle regulation, apoptosis and differentiation, involved in the late stage of mitosis, embryonic development, DNA stress response, and is likely to participate in the occurrence of tumors.^[6–9]^ As an epithelial transcription inhibitor, amplification, overexpression or deletion of E2F7 can be observed in many malignant tumors, and it can affect tumor differentiation, proliferation and metastasis by interacting with different downstream targets. E2F7 is abnormally expressed in glioma,^[10,11]^ colon cancer^[12–14]^ and breast cancer,^[15,16]^ and has an important influence on the occurrence and development of a variety of tumors.

In view of this, we conducted this study to explore the expression of E2F7 in lung adenocarcinoma (LUAD) and analyze its correlation with clinical parameters, diagnostic and prognostic value of LUAD patients.

## 2. Materials and Method

### 2.1. Patient data set

E2F7 mRNA expression data (including 594 samples, data format: FPKM) and clinical characteristics data are downloaded from the TCGA database. The data for pan-cancer analysis is from UCSC XENA (https://xenabrowser.net/datapages/). It is the RNAseq data in TPM format of TCGA and GTEx that has been uniformly converted by the Toil process. Inclusion criteria: 1. Sufficient survival information; 2. Definite gene expression value.

All our data come from public databases such as GEO and TCGA. The patients involved in the database have obtained ethical approval. Our research is based on open-source data and therefore does not require ethics committee approval for the study.

### 2.2. Statistical analysis

The median of E2F7 expression was selected as the critical value, and the Wilcoxon signed rank test was used to test the differential expression of E2F7 in LUAD and normal tissues, and the results were displayed by box plots. Wilcoxon rank sum test and Dunn's test were used to testing whether the expression of E2F7 is related to clinical features in LUAD. Kaplan-Meier analysis was performed to compare the differences in OS and DSS between E2F7 high and low expression groups, and to draw survival curves.^[17]^ The pROC package and the ggplot2 package are used to study the role of E2F7 prognosis and draw the ROC curve, where AUC represents the diagnostic value. Univariate Cox regression analysis was used to screen potential prognostic factors, and multivariate Cox regression was used to verify the independent predictive value of multiple indicators including E2F7 for prognosis. The rms package and survival package are used to draw nomograms to show the relationship between various variables and survival rates. The clusterProfiler package and the org.Hs.eg.db package are used for the enrichment analysis of GO and KEGG.^[18]^ The clusterProfiler package and the ggplot2 package are used to perform GSEA enrichment analysis and plotting. In addition, we used an independent GEO data set (GSE50081) for external verification. The difference in the expression of E2F7 in pan-tumor and normal tissues is verified in UCSC XENA (https://xenabrowser.net/datapages/)^[19]^ and Timmer database (https://cistrome.shinyapps.io/timmer/). All statistical analysis uses R statistical software (version 3.6.3).

## 3. Result

### 3.1. Baseline characteristics of included patients

A total of 535 patients diagnosed with lung adenocarcinoma were included in this study, and the data of these patients were all obtained through the TCGA data portal. The detailed clinical characteristics are shown in Table 1. Among the included patients, 249 were males (46.5%) and 286 were females (53.5%). Regarding the TNM staging of lung cancer: 294 patients were stage I, 123 patients were stage II, 84 patients were stage III, and 26 patients were stage IV. The median age of patients in the E2F7 high and low expression group was 65 and 67 years, respectively, and the results were not statistically different (*P* = .346). Regarding surgical treatment: the number of patients undergoing R0, R1, and R2 resection was 355, 13, and 4, respectively, and there was no significant difference between the groups (*P* = .186). In gender (*P* = .041), number pack year smoked (*P* = .018), M stage (*P* = .034) and OS event (*P* = .041), there are significant differences between the 2 groups.

**Table 1:** Main characteristics of LUAD patients.

Characteristic	levels	Low expression of E2F7	High expression of E2F7	*P*
n		267	268	
Age, n (%)	<=65	121 (23.4%)	134 (26%)	.291
	>65	137 (26.6%)	124 (24%)	
Gender, n (%)	Female	155 (29%)	131 (24.5%)	.041
	Male	112 (20.9%)	137 (25.6%)	
Race, n (%)	Asian	2 (0.4%)	5 (1.1%)	.359
	Black or African American	25 (5.3%)	30 (6.4%)	
	White	208 (44.4%)	198 (42.3%)	
Smoker, n (%)	No	43 (8.3%)	32 (6.1%)	0.169
	Yes	214 (41.1%)	232 (44.5%)	
number_pack_years_smoked, n (%)	<40	102 (27.6%)	86 (23.3%)	0.018
	>=40	75 (20.3%)	106 (28.7%)	
Pathologic stage, n (%)	Stage I	152 (28.8%)	142 (26.9%)	0.210
	Stage II	61 (11.6%)	62 (11.8%)	
	Stage III	39 (7.4%)	45 (8.5%)	
	Stage IV	8 (1.5%)	18 (3.4%)	
T stage, n (%)	T1	97 (18.2%)	78 (14.7%)	0.336
	T2	135 (25.4%)	154 (28.9%)	
	T3	24 (4.5%)	25 (4.7%)	
	T4	10 (1.9%)	9 (1.7%)	
N stage, n (%)	N0	178 (34.3%)	170 (32.8%)	0.787
	N1	49 (9.4%)	46 (8.9%)	
	N2	33 (6.4%)	41 (7.9%)	
	N3	1 (0.2%)	1 (0.2%)	
M stage, n (%)	M0	188 (48.7%)	173 (44.8%)	0.034
	M1	7 (1.8%)	18 (4.7%)	
Anatomic neoplasm subdivision, n (%)	Left	99 (19%)	106 (20.4%)	0.640
	Right	160 (30.8%)	155 (29.8%)	
Anatomic neoplasm subdivision2, n (%)	Central Lung	32 (16.9%)	30 (15.9%)	0.540
	Peripheral Lung	58 (30.7%)	69 (36.5%)	
Residual tumor, n (%)	R0	176 (47.3%)	179 (48.1%)	0.186
	R1	6 (1.6%)	7 (1.9%)	
	R2	0 (0%)	4 (1.1%)	
Primary therapy outcome, n (%)	PD	27 (6.1%)	44 (9.9%)	0.109
	SD	21 (4.7%)	16 (3.6%)	
	PR	3 (0.7%)	3 (0.7%)	
	CR	177 (39.7%)	155 (34.8%)	
OS event, n (%)	Alive	183 (34.2%)	160 (29.9%)	0.041
	Dead	84 (15.7%)	108 (20.2%)	
DSS event, n (%)	Alive	198 (39.7%)	181 (36.3%)	0.079
	Dead	51 (10.2%)	69 (13.8%)	
PFI event, n (%)	Alive	162 (30.3%)	147 (27.5%)	0.202
	Dead	105 (19.6%)	121 (22.6%)	
Age, meidan (IQR)		67 (59, 72)	65 (59, 72)	0.346

CR = complete response, DSS = Disease Free Survival, OS = overall survival, PD = progressive disease, PFI = Progression Free Interva, PR = partial response, SD = stable disease.

### 3.2. High expression of E2F7 in LUAD

**Figure 1.**
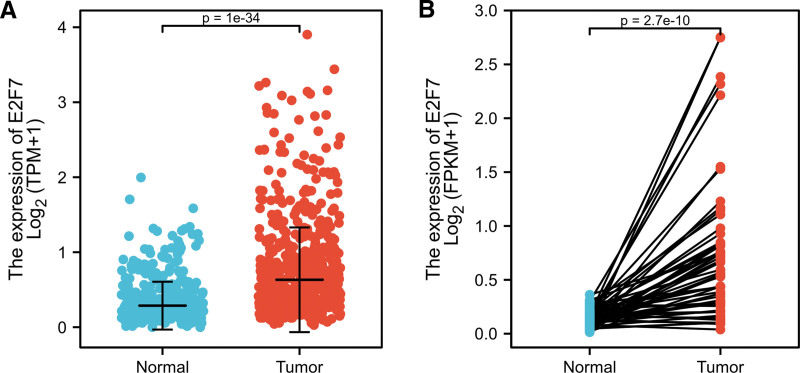
E2F7 expression in LUAD tissues. A. E2F7expression in normal and tumor tissue. B. E2F7 expression in paired tissue.

We compared the expression levels of E2F7 in LUAD and normal lung tissues. Taking the median of the gene expression level of CCNA2 as the cutoff value, the patients were divided into high expression group and low expression group. The results of the study on unpaired samples showed that the expression of E2F7 in LUAD was higher than that of normal lung tissue (*P* = 1e-34) (Fig. 1A). In the paired samples of LUAD and normal lung tissue, this conclusion was verified. (*P* = 2.7e-10) (Fig. 1B).

### 3.3. E2F7 expression and clinical characteristics

**Figure 2.**
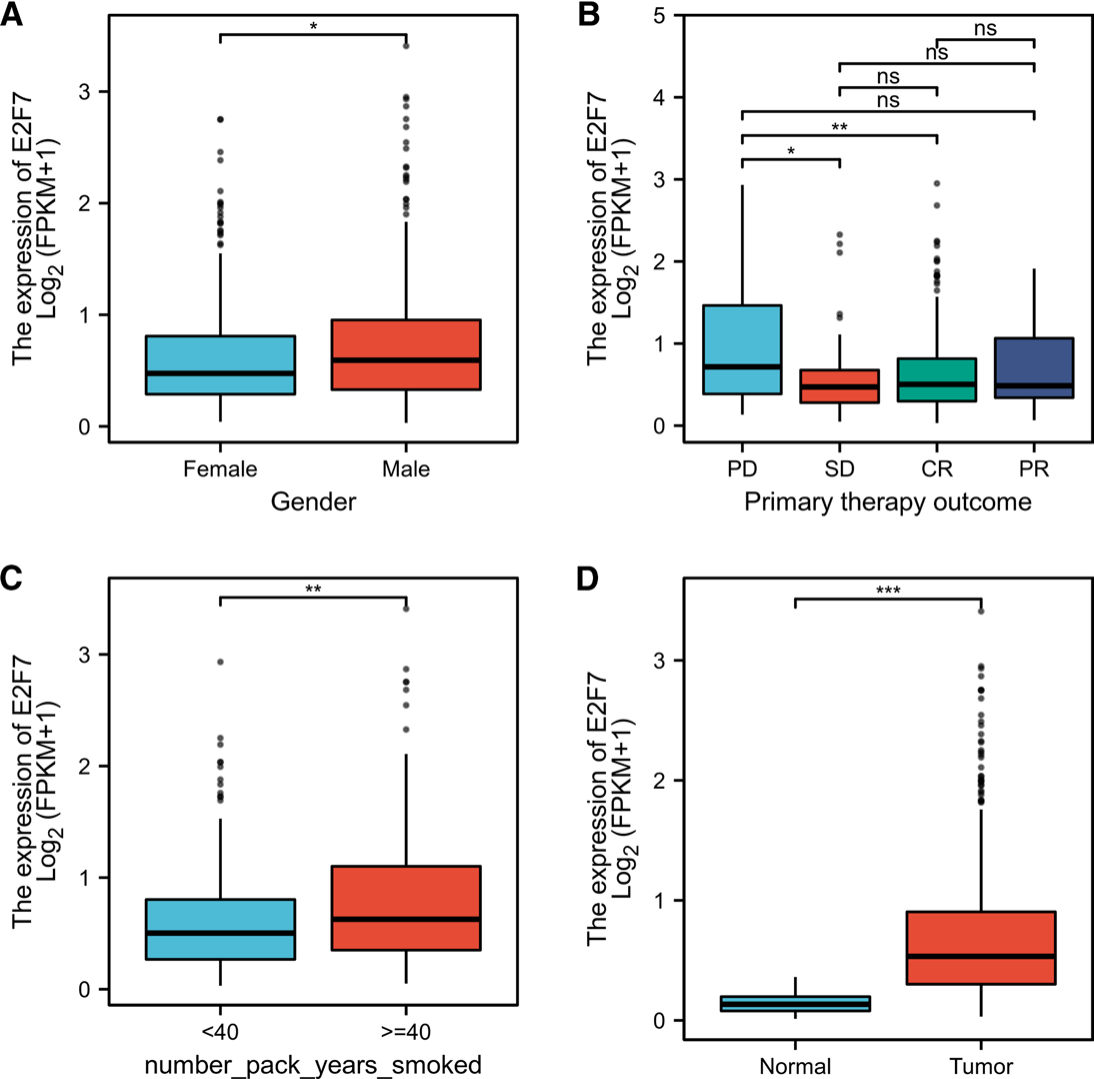
Box plot of E2F7 expression of LUAD patients according to different clinical characteristics. A. Gender. B. Primary therapy outcome. C. Number pack years smoked. D. Cancer status. ns, *P* ≥ .05; ^*^P < .05; ^**^P < .01; ^***^P < .001.

The logistic regression analysis results of the correlation between E2F7 expression level and clinical characteristics are summarized in Table 2. The high expression of E2F7 was significantly correlated with gender (*P* = .034), pathologic stage (*P* = .046) and M stage (*P* = .025). As shown in Figure 2, the Mann-Whitney U test results verify the correlation between E2F7 expression and gender (*P* = .029) and the number pack-years smoked (*P* = .002). The results of multiple hypothesis test (Dunn's test) using Bonferroni method to correct the significance level show that the difference between SD and PD (*P*.adj = .037), CR and PD (*P*.adj = .001) was statistically significant. The same result appeared in the comparison of tumor and normal tissue (*P* < .001).

**Table 2:** Logistic analysis of the association between E2F7 expression and clinical characteristics.

Characteristics	Total (N)	OR (95%CI)	*P* value
Age (>65 vs <=65)	516	0.817 (0.578–1.154)	.253
Gender (Male vs Female)	535	1.447 (1.030–2.038)	.034
Race (Asian&White vs Black or African American)	468	0.806 (0.455–1.415)	.453
Smoker (Yes vs No)	521	1.457 (0.892–2.402)	.135
Pathologic stage (Stage IV vs Stage I)	320	2.408 (1.047–6.029)	.046
T stage (T2&T3&T4 vs T1)	532	1.383 (0.963–1.993)	.080
N stage (N1 vs N0)	443	0.983 (0.623–1.548)	.941
M stage (M1 vs M0)	386	2.794 (1.186–7.337)	.025
Anatomic neoplasm subdivision (Left vs Right)	520	1.105 (0.777–1.572)	.577
Anatomic neoplasm subdivision2 (Central Lung vs Peripheral Lung)	189	0.788 (0.428–1.448)	.443
Residual tumor (R1&R2 vs R0)	372	1.803 (0.671–5.331)	.256
Primary therapy outcome (PD&SD vs CR&PR)	446	1.424 (0.922–2.208)	.112

CR = complete response, PD = progressive disease, PR = partial response, SD = stable disease.

### 3.4. E2F7 high expression is an independent prognostic risk factor

**Figure 3.**
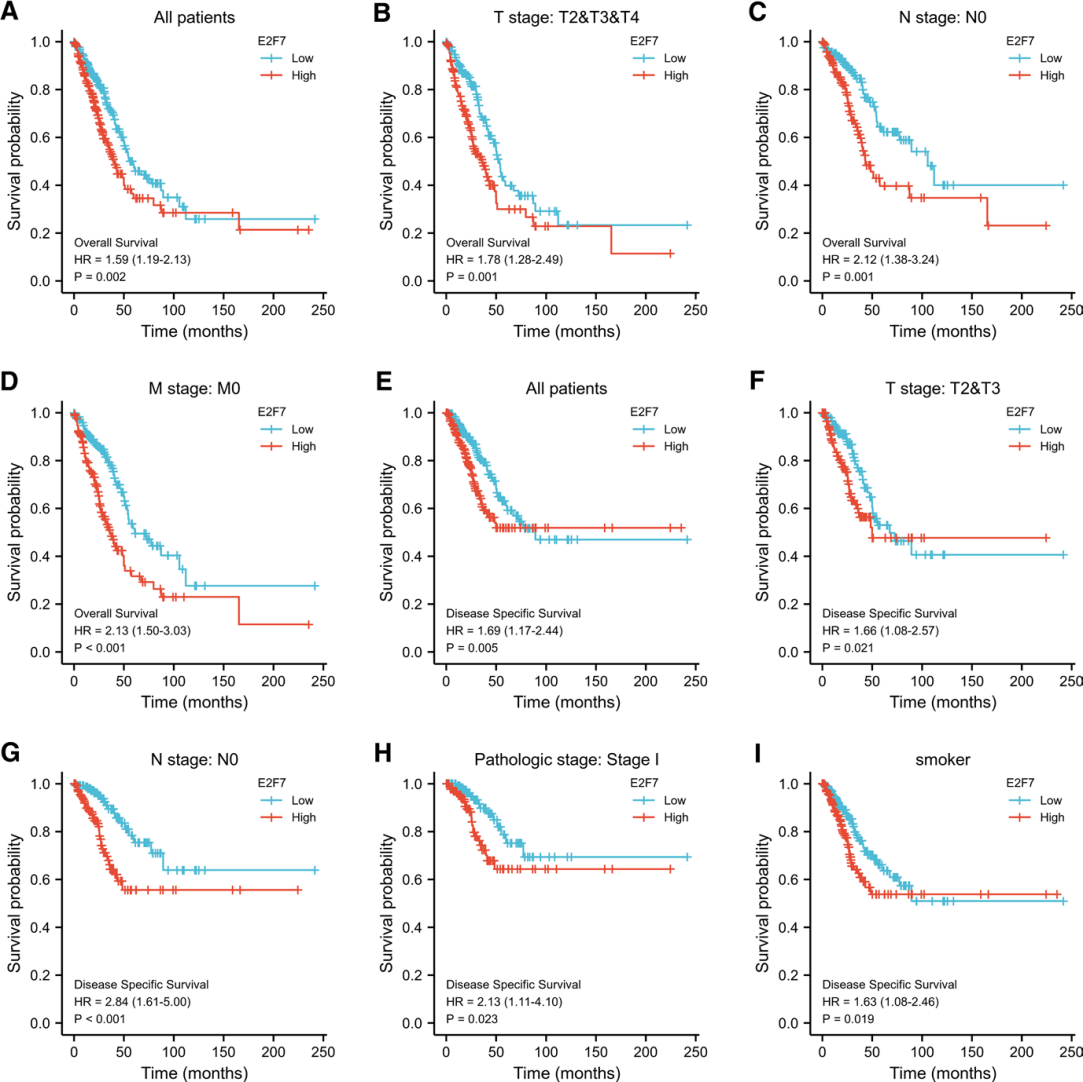
Kaplan-Meier curve for survival in LUAD. A. OS of all patients. B. OS of T2/T3/T4. C. OS of N0. D. OS of M0. E. DSS of all patients. F. DSS of T2/T3. G. DSS of N0. H. DSS of stage I. DSS of smoker.

Kaplan-Miere survival analysis of all adenocarcinoma patients showed that high expression of E2F7 was associated with shorter OS (*P* = .002) (Fig. 3A). The results of subgroup analysis showed that in patients with T2/T3/T4 (*P* = .001) (Fig. 3B), N0 (*P* = .001) (Fig. 3C), M0 (*P* < 0.001) (Fig. 3D), E2F7 was highly expressed Significantly related to shorter OS. In terms of DSS (*P* = .005), E2F7 showed similar results (Fig. 3E), at T2/T3 (*P* = .021) (Fig. 3F) The prognostic value of E2F7 in the subgroups of, N0 (*P* < .001) (Fig. 3G), M0 (*P* = .023) (Fig. 3H) and smoker (*P* = .019) (Fig. 3I) was also verified.

**Figure 4.**
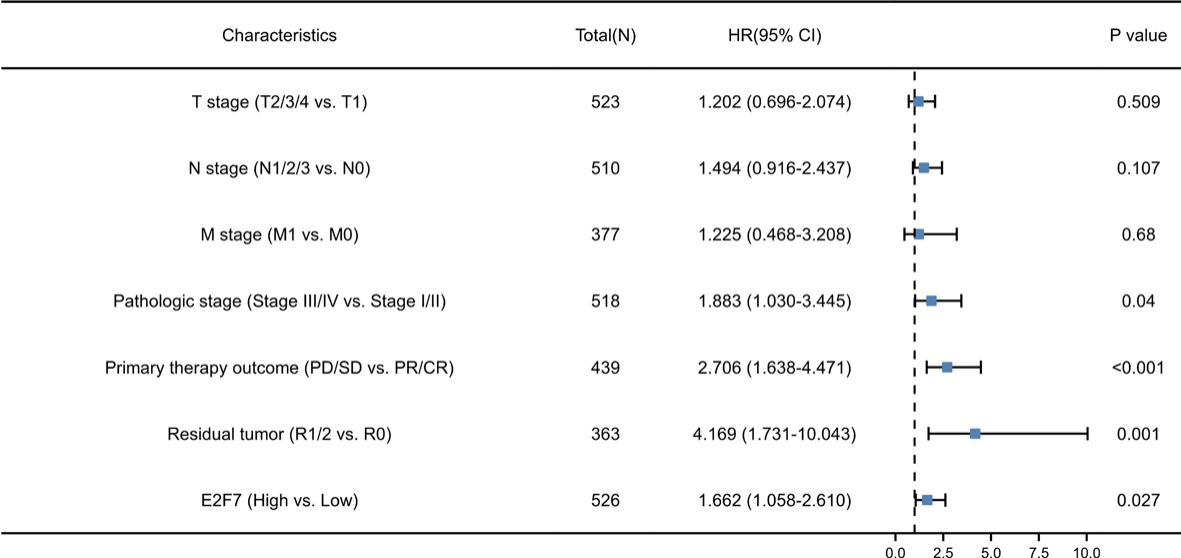
Forest plot of the multivariate Cox regression analysis of OS in LUAD.

Univariate Cox regression results show T stage (*P* = .002), N stage (*P* < .001), M stage (*P* = .006), pathologic stage (*P* < .001), primary therapy outcome (*P* < .001), residual tumor (*P* < .001) and E2F7 (*P* = .002) were significantly related to poor prognosis. Further multivariate Cox analysis confirmed that pathologic stage (*P* = .040), primary therapy outcome (*P* < .001), residual tumor (*P* = .001) and E2F7 (*P* = .027) are independent factors affecting the prognosis of LUAD patients (Fig. 4, Table 3).

**Table 3:** Univariate and multivariate Cox regression analysis of the relationship between clinical characteristics and overall survival.

Characteristics	Total (N)	Univariate analysis		Multivariate analysis	
		Hazard ratio (95% CI)	*P* value	Hazard ratio (95% CI)	*P* value
T stage (T2&T3&T4 vs T1)	523	1.728 (1.229–2.431)	**.002**	1.202 (0.696–2.074)	.509
N stage (N1&N2&N3 vs N0)	510	2.601 (1.944–3.480)	**<.001**	1.494 (0.916–2.437)	.107
M stage (M1 vs M0)	377	2.136 (1.248–3.653)	**.006**	1.225 (0.468–3.208)	.680
Age (>65 vs <=65)	516	1.223 (0.916–1.635)	.172		
Gender (Male vs Female)	526	1.070 (0.803–1.426)	.642		
Pathologic stage (Stage III&Stage IV vs Stage I&Stage II)	518	2.664 (1.960–3.621)	**<.001**	1.883 (1.030–3.445)	**.040**
Primary therapy outcome (PD&SD vs PR&CR)	439	2.653 (1.888–3.726)	**<.001**	2.706 (1.638–4.471)	**<.001**
Residual tumor (R1&R2 vs R0)	363	3.879 (2.169–6.936)	**<.001**	4.169 (1.731–10.043)	**.001**
Anatomic neoplasm subdivision (Right vs Left)	512	1.037 (0.770–1.397)	.810		
Smoker (Yes vs No)	512	0.894 (0.592–1.348)	.591		
Race (Black or African American vs White&Asian)	468	0.698 (0.422–1.157)	.163		
E2F7 (High vs Low)	526	1.594 (1.193–2.129)	**.002**	1.662 (1.058–2.610)	**.027**
number_pack_years_smoked (<40 vs >=40)	363	0.932 (0.654–1.328)	.697		

CR = complete response, PD = progressive disease, PR = partial response, SD = stable disease.

### 3.5. The diagnostic value of E2F7

**Figure 5.**
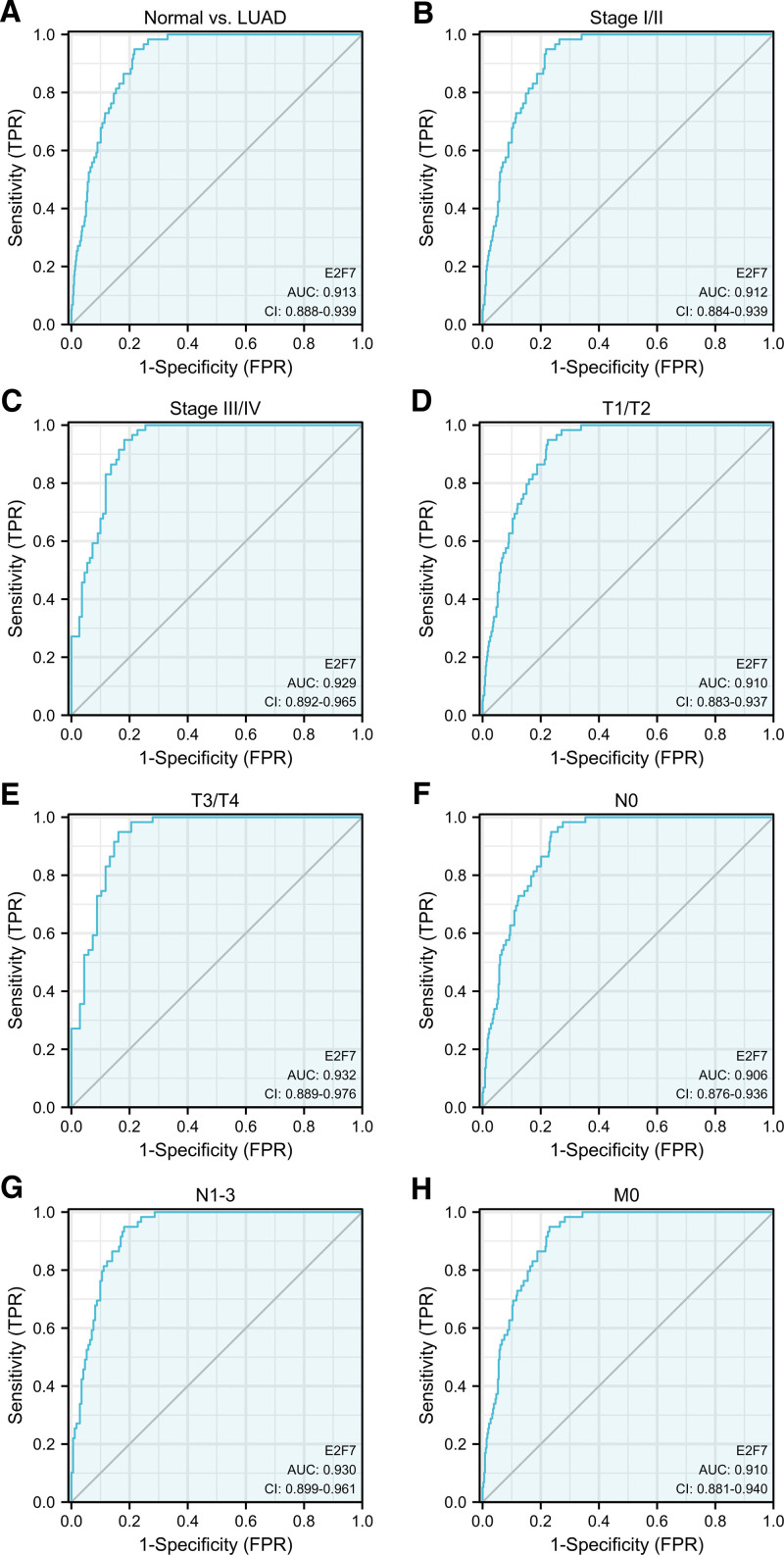
ROC curve of E2F7 expression in LUAD. A. Normal vs LUAD. B. Stage I/II. C. Stage III/IV. D. T1/T2. E. T3/T4. F. N0. G. N1-3. H. M0.

We performed ROC curve analysis on the expression data of E2F7, and the results showed that this index has a high diagnostic value for patients with LUAD (AUC = 0.913, 95%CI: 0.888–0.939) (Fig. 5A). Further subgroup analysis verified its diagnostic value in stage I/II (AUC = 0.912) (Fig. 5B), stage III/IV (AUC = 0.929) (Fig. 5C), T1/T2 (AUC = 0.910) (Fig. 5D), T3/T4 (AUC = 0.932) (Fig. 5E), N0 (AUC = 0.906) (Fig. 5F), N1–3 (AUC = 0.930) (Fig. 5G) and M0 (AUC = 0.910) (Fig. 5H).

**Figure 6.**
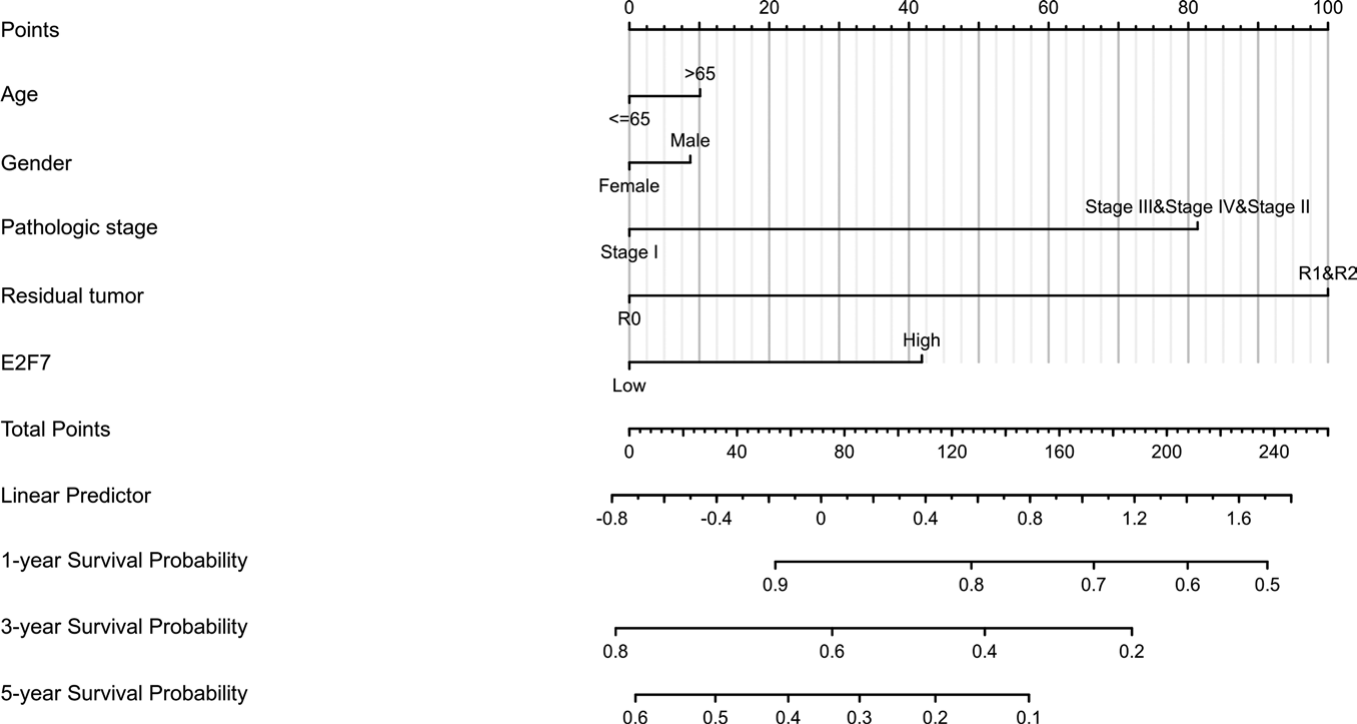
Nomogram for predicting the probability of LUAD patients with 1-, 3-, 5-year OS.

Due to its high diagnostic value, we combined E2F7 with clinical variables widely considered to be related to prognosis to construct a nomogram to predict the 1-, 3-, and 5-year survival probability (Fig. 6).

### 3.6. E2F7 related signal pathways

We performed GO/KEGG enrichment analysis on E2F7. Under the conditions of *P*.adj < 0.1 and *q* value<0.2, there are 6 BPs, 12 CCs, 1 MF, and KEGG 2 signal pathways (Table 4).

**Table 4:** Gene sets enriched in the high E2F7 expression phenotype.

ONTOLOGY	ID	Description	Gene Ratio	Bg Ratio	*P* value	*P*.adjust	FDR q-value
BP	GO:0000353	formation of quadruple SL/U4/U5/U6 snRNP	4/300	12/18670	2.92e-05	0.020	0.020
BP	GO:0000365	mRNA trans splicing, via spliceosome	4/300	12/18670	2.92e-05	0.020	0.020
BP	GO:0045291	mRNA trans splicing, SL addition	4/300	12/18670	2.92e-05	0.020	0.020
BP	GO:0007389	pattern specification process	20/300	446/18670	3.85e-05	0.020	0.020
BP	GO:0000244	spliceosomal tri-snRNP complex assembly	5/300	26/18670	5.17e-05	0.022	0.022
CC	GO:0015030	Cajal body	10/309	77/19717	3.35e-07	7.95e-05	7.83e-05
CC	GO:0072588	box H/ACA RNP complex	4/309	10/19717	1.15e-05	0.001	0.001
CC	GO:0005732	small nucleolar ribonucleoprotein complex	5/309	28/19717	6.69e-05	0.005	0.005
CC	GO:0097525	spliceosomal snRNP complex	7/309	99/19717	9.43e-04	0.050	0.050
CC	GO:0030532	small nuclear ribonucleoprotein complex	7/309	105/19717	0.001	0.050	0.050
MF	GO:0001228	DNA-binding transcription activator activity, RNA polymerase II-specific	17/223	439/17697	4.37e-05	0.012	0.012
KEGG	hsa05034	Alcoholism	8/75	187/8076	3.19e-04	0.037	0.036
KEGG	hsa05322	Systemic lupus erythematosus	6/75	136/8076	0.002	0.092	0.089

BP = biological process, CC = cellular component, KEGG = Kyoto Encyclopedia of Genes and Genomes, MF = molecular function.

*P*.adj<.05 and FDR q-value<.2 were considered as significantly enriched.

**Figure 7.**
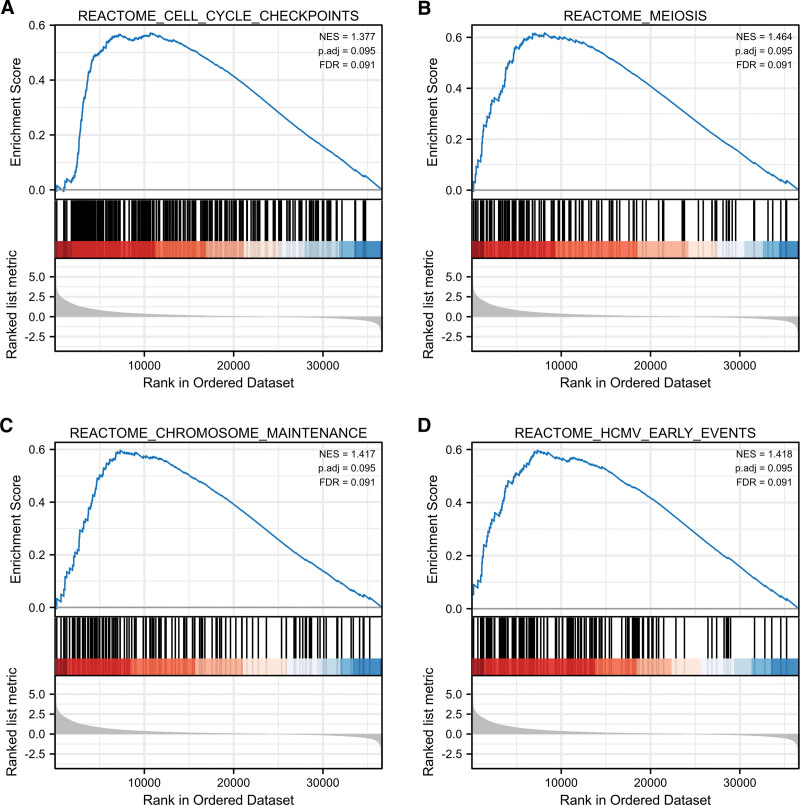
Enrich plots from GSEA. The possible biological processes or signaling pathways of E2F7.

We performed GSEA on the data set of high and low expression of E2F7 to determine the differentially activated signaling pathways in LUAD. A total of 39 data sets satisfy FDR (*q* value) <0.25 and *P*.adjust < 0.05. Cell cycle checkpoints, DNA damage telomere stress-induced senescence, DNA methylation, chromosome maintenance and mitotic prophase and other pathway-related genes showed enrichment in the high E2F7 expression group (Fig. 7).

### 3.7. Verification through other independent external databases

**Figure 8.**
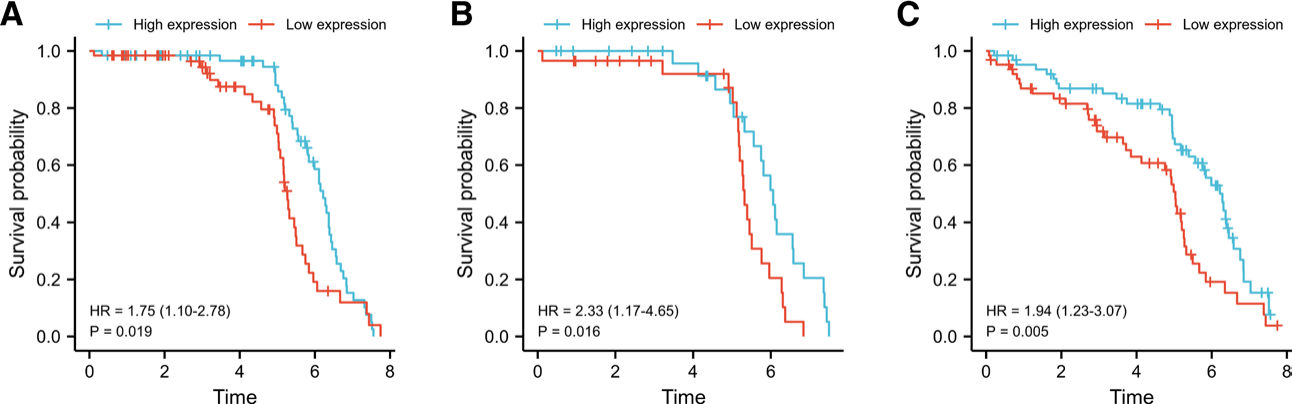
Kaplan-Meier curve for survival in LUAD patients in the validation datasets GSE50081. A. OS of all patients. B. OS of ex-smoker. C. Disease-free survival (DFS) of all patients.

We used an independent GEO dataset (GSE50081) containing 127 LUAD patients to further verify the above results. The results of the Kaplan-Meier survival analysis confirmed the prognostic value of E2F7 for LUAD patients (Fig. 8A–C).

**Figure 9.**
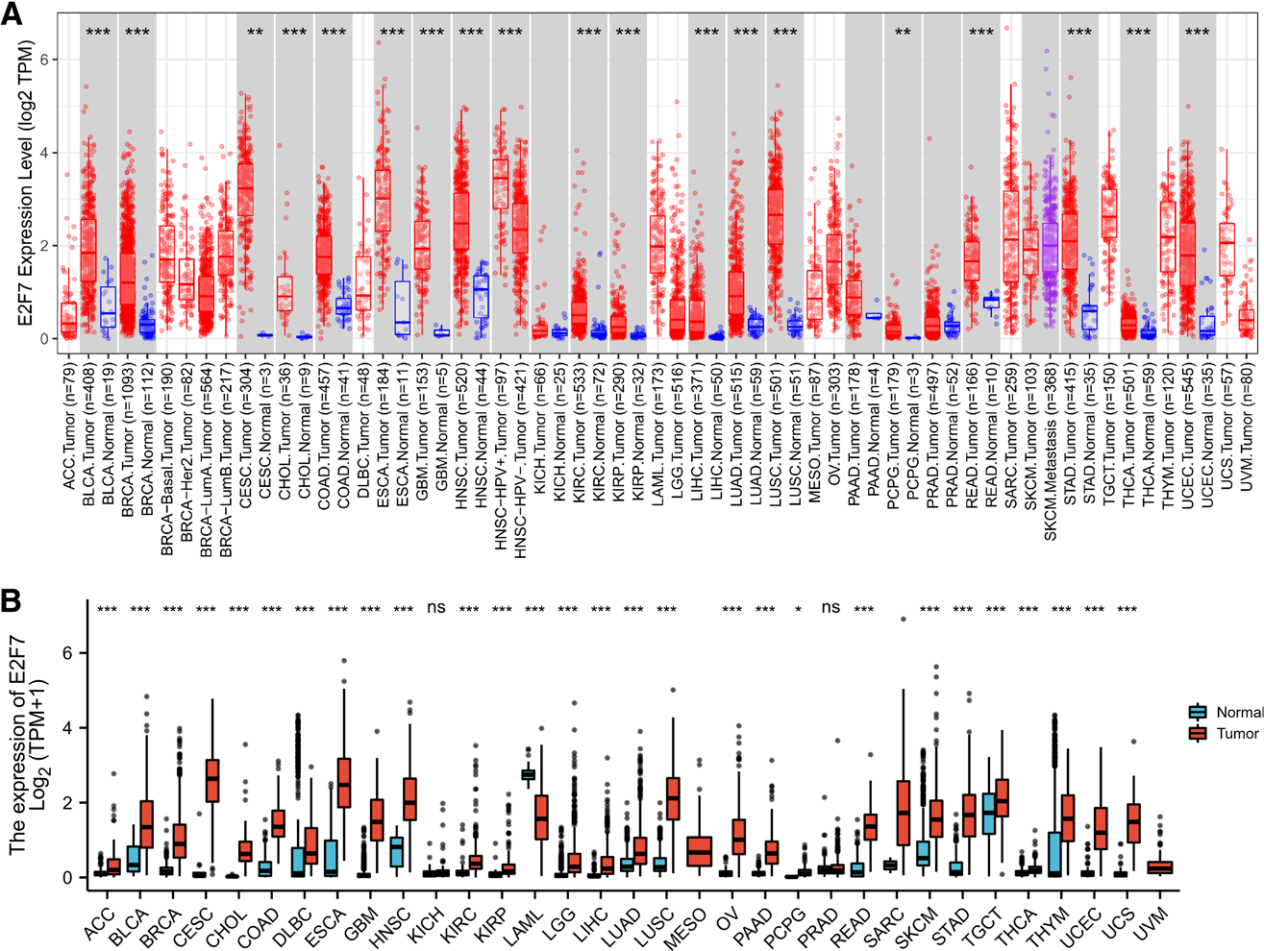
Expression analysis of E2F7 in different types of human tumors. A. Timmer databases. B. TCGA+ GTEx.

We used the Timmer database to perform pan-tumor E2F7 expression analysis and showed that E2F7 is highly expressed in a variety of solid tumors including LUAD (Fig. 9A). We also integrated the pan-tumor analysis of the two databases of TCGA and GTEx and reached similar conclusions (Fig. 9B).

## 4. Discussion

In our study, the expression of E2F7 in many tumors including LUAD was higher than normal, and its expression level was higher in men and patients greater than 40 number pack-year, and it was related to the primary therapy outcome of disease, that is It is said that patients with the progressive disease have higher expression of E2F7. Subsequent survival analysis also showed that high expression of E2F7 is an independent risk factor for OS, and it has a high diagnostic value. This provides a basis for E2F7 to judge the prognosis of LUAD patients in future clinical work. Genes related to cell cycle checkpoints, DNA damage, telomere stress-induced senescence, DNA methylation, chromosome maintenance, and mitogenic pathways showed significant enrichment in the E2F7 high expression group, suggesting that E2F7 affects lung adenocarcinoma The potential mechanism of occurrence and development provides an important reference for further exploration of its mechanism through experiments in the future.

The occurrence and development of malignant tumors is a complex process involving multiple genes and their expressed proteins. Transcription is the beginning of gene expression and is strictly regulated by transcription factors (TFs) and its cofactors, RNA polymerase, and chromatin-modifying proteins.^[6]^

E2Fs are an important family of transcription factors, which have been confirmed to be involved in the process of cell proliferation,^[20–23]^ differentiation,^[24–26]^ apoptosis,^[27–30]^ cycle regulation^[31,32]^ and DNA damage response.^[33,34]^ So far, a total of 8 family members have been discovered (E2F1-E2F8). According to their different functions, E2Fs are divided into transcription activators (E2F1–3) and transcription repressors (E2F4–8), and according to their structure, they are divided into typical E2Fs (E2F1–6) and atypical E2Fs (E2F7–8). The clinical value of many E2Fs members in the diagnosis and treatment of many solid tumors has been affirmed.^[35–38]^

E2F7 is different from the typical E2Fs members in that it binds to DNA in a non-DP protein way to play a transcriptional inhibitory effect.^[4,39]^ Studies have shown that E2F7 can inhibit cell proliferation by inhibiting the transcription of proliferation-related miRNAs.^[40]^ However, in recent years, more and more studies have shown that E2F7 plays a role in promoting tumor occurrence and development in tumors. Chu et al. reported that the overexpression of E2F7 in breast cancer can inhibit miR-15a/16 transcription, cause Cyclin E1 and Bcl-2 to participate in tumor invasion and metastasis, and increase the resistance of breast cancer cells to tamoxifen.^[15]^

In our study, the expression of E2F7 in a variety of solid tumors was analyzed through the Timmer database and UCSC XENA. The results showed that E2F7 is highly expressed in LUAD, lung squamous cell carcinoma (LUSC), esophageal squamous cell carcinoma (ESCA) and other solid tumors.

In previous existing studies, there is no content about the prognostic value of E2F7 expression in LUAD patients. In this study, the diagnostic value of E2F7 was analyzed on the TCGA database by means of bioinformatics analysis. Kaplan-Meier survival analysis showed that high expression of E2F7 was associated with shorter OS and DSS, and this conclusion was verified in the GEO dataset. Multivariate Cox analysis further confirmed that the expression of E2F7 is independently related to OS of patients with LUAD. Other clinical features, such as local advanced stage, lymph node metastasis, distant metastasis, later TNM staging, and the degree of surgical resection are closely related, and are also related to poor prognosis. We further constructed a nomogram of the prognosis of LUAD patients based on clinical variables and the expression of E2F7, which provided a basis for clinicians to predict the survival rate of individual patients.

The mechanism by which E2F7 mediates the development of tumors is not completely clear. It may promote tumor proliferation, differentiation, infiltration and metastasis through the following methods: (1) E2F7 up-regulates Beclin-1 and mediates autophagy induced by miR-129 Trigger autophagy flux^[10]^; (2) E2F7 increases the expression level of vimentin, reduces the expression of E-cadherin protein, and promotes the EMT process^[41–43]^; (3) As the transcriptional activators of VEGFA, E2F7 cooperates with HIF-1α to induce the transcription of VEGFA and promote blood vessel Generation^[44]^; (4) Induce the transcription of collagen and calcium-binding domains and Flt to promote the generation of lymphatic vessels.^[35,45]^ Our study found that the expression of E2f7 is related to pathways such as cell cycle checkpoints, DNA damage telomere stress-induced senescence, DNA methylation, chromosome maintenance and mitotic prophase. Our research results are related to the above-mentioned mechanisms, but these mechanisms need further research to confirm.

Although our study provides a new method to explore the relationship between E2F7 and the prognosis of lung adenocarcinoma, it still has many limitations. First of all, although we have adopted the GEO database to verify the results of the TCGA database analysis, the study object is still only patients in the public database, which will lead to bias. Secondly, due to the limited sample size and clinical indicator, our research conclusions need to be further confirmed by a large sample of research. Finally, we need further experiments to explore the role of E2F7 in tumor progression and its mechanism of affecting tumors.

In short, high expression of E2F7 is an independent risk factor for OS in LUAD patients, and has a high diagnostic value. cell cycle checkpoints, DNA damage telomere stress-induced senescence, DNA methylation, chromosome maintenance and mitotic prophase may be the key pathways through which LUAD is regulated by E2F7.
